# A case of Cornelia de Lange syndrome from Sudan

**DOI:** 10.1186/1471-2431-7-6

**Published:** 2007-01-29

**Authors:** Mona Ellaithi, David Gisselsson, Therese Nilsson, Atif Elagib, Imad Fadl-Elmula, Mashair Abdelgadir

**Affiliations:** 1 The Orchids Organization for Children with Special Needs, Khartoum, Sudan; 2 Institute of Endemic Diseases, University of Khartoum, Khartoum, Sudan; 3 Department of Clinical Genetics, Lund University, Lund, Sweden; 4 Tropical Medical Research Institute, Khartoum, Sudan; 5 Faculty of Medical Laboratory Sciences, Al Neelain University, Khartoum, Sudan; 6 Khartoum Teaching Hospitals, Khartoum, Sudan

## Abstract

**Background:**

Brachmann de Lange syndrome (BDLS) is a multiple congenital anomaly syndrome characterized by a distinctive facial appearance, prenatal and postnatal growth deficiency, psychomotor delay, behavioral problems, and malformations of the upper extremities.

**Case presentation:**

Here we present for the first time a case of BDLS from Sudan, a 7-month-old female infant, who was referred as a case of malnutrition. The patient was from a Sudanese western tribe. Clinical investigation showed that the child was a classical case of BDLS, but with some additional clinical findings not previously reported including crowded ribs and tied tongue.

**Conclusion:**

Reporting BDLS cases of different ethnic backgrounds could add nuances to the phenotypic description of the syndrome and be helpful in diagnosis.

## Background

Brachmann de Lange Syndrome (BDLS) [1], also known as Cornelia de Lange syndrome [2] or Brachmann Cornelia de Lange syndrome is a dominantly inherited multi-system developmental disorder characterized by growth and cognitive retardation, abnormalities of the upper limbs, gastroesophageal dysfunction, ophthalmologic and genitourinary anomalies, hirsutism, characteristic facial features, pyloric stenosis, congenital diaphragmatic hernias, cardiac septal defects, and hearing loss. Autism and self-injurious tendencies also frequently occur. The prevalence of the syndrome is estimated to be as high as 1 in 10,000 [3]. Facial findings, including characteristic eyebrows (neat, well-defined, and arched), long philtrum, thin lips, and crescent-shaped mouth, are the most important diagnostic features of BDLS. This combination of facialanomalies is absent in postpubertal males but not in postpubertal females [4].

Van Allen and colleagues in 1993, proposed a classification system for BDLS. Type I, or classic BDLS, patients have the characteristic facial and skeletal changes of the diagnostic criteria established by Preus and Rex in 1983 [5,6]. They have prenatal growth deficiency, moderate-to-profound psychomotor retardation, and major malformations, which result in severe disability or death. Type II, or mild BDLS, patients have similar facial and minor skeletal abnormalities to those seen in type I; however, these changes may develop with time or may be only partially expressed. They have mild-to-borderline psychomotor retardation, less severe pre- and postnatal growth deficiency, and the absence of (or less severe) major malformations. Type III, or phenocopy BDLS, includes patients who have phenotypic manifestations of BDLS that are causally related to chromosomal alterations or teratogenic exposures. Allanson and colleagues in 1997 concluded that, in the mild phenotype, the characteristic facial appearance may not appear until 2 to 3 years of age, while it is always present at birth in the classic phenotype. They also noted that the characteristic facial appearance decreased with time in the mild phenotype. Craniofacial pattern profiles showed that both type I and type II groups had microbrachycephaly, but that the dimensions of the mild group were somewhat closer to normal [7].

In rare cases, constitutional chromosomal imbalances can simulate the features of BDLS. For example, DeScipio and colleagues in 2005 reported 2 half-sibs with clinical features suggestive of BDLS and an unbalanced chromosomal rearrangement, der(3)t(3;12)(p25.3;p13.3), inherited from a balanced translocation in their unaffected mother [8]. The sibs had many features consistent with BDLS, including microcephaly, growth retardation, mental retardation, hirsutism, synophrys, anteverted nares, single palmer creases, and syndactyly of toes 2 and 3, but also showed significant clinical overlap with del(3)(p25) syndrome. However, the vast majority of BDLS cases have normal karyotypes by chromosome banding. Ptacek and colleagues in 1963 suggested dominant inheritance [9], but Opitz in 1971 and 1985 thought recessive inheritance more likely [10,11]. Robinson and Jones in 1983 supported the conclusion that BDLS is autosomal dominant and that the sporadic occurrence in most cases reflects the genetic lethality of the disorder. Their cases were a severely affected 5-month-old boy and his mildly affected 24-year-old mother [12]. Although most cases are sporadic, rare families with multiple affected sibs have been described [13]. Fryns and colleagues in 1987 reported 2 infant brothers with a severe form of the syndrome. They died at the ages of 3 months and 3 weeks, respectively. The parents were normal, and prometaphase chromosome studies failed to show any abnormality [14]. In the same year Naguib and colleagues described an Arab family with phenotypically normal first-cousin parents and 2 offspring showing variable features of this disorder. The proband had apparently normal chromosomes and died at the age of 3 months. His sister was less severely affected and lived for 6 years. The authors suggested recessive inheritance [15]. McConnell and colleagues in 2003 supported the dominant inheritance theory, reporting a family with a neonate affected by classical BDLS, an affected mother, and a probably affected maternal grandmother [16].

Krantz and colleagues in 2004, and also Tonkin in 2004 performed genome-wide linkage exclusion mapping in 12 BDLS families and identified a locus in chromosome band 5p13. This locus mapped close to both a translocation breakpoint and a small *de novo *deletion associated with BDLS. Further mapping of the 5p13 translocation breakpoint showed disruption of the *NIPBL *gene [3,17]. Recently, Yan and colleagues identified 13 *NIPBL *mutations identified in 28 unrelated Polish BDLS patients. Mutation-positive patients were more severely affected in comparison to mutation negative individuals with respect to weight, height, and mean head circumference at birth, facial dysmorphism and speech impairment. Analyses of combined data from this and the two previous studies revealed that the degree of growth retardation, developmental delay and limb defects showed significant differences between patients with and without mutations and between patients with missense and truncating mutations, whereas only a portion of these features differed significantly in any individual study [18]. Bhuiyan and colleagues in 2006 described genotype-phenotype correlations in 39 patients. They found mutations of NIPBL in 56% of the patients. Truncating mutations generally caused a more severe phenotype, but this correlation was not absolute [19]. Musio and colleagues (2006) recruited 53 unrelated and 4 related individuals with a diagnosis of BDLS, encompassing the entire spectrum of phenotypes. They found pathogenic *NIPBL *mutations in 24 of them, whereas the remaining 33 cases did not have any *NIPBL *alterations. Of these 33 individuals, there was only 1 instance of familial occurrence, with 2 male sibs, their mother, and a first cousin affected. Involvement of *NIPBL *was excluded in this family, but the affected individuals were found to carry a 3-bp deletion in the *SMC1L1 *gene in Xp11. In addition, a sporadic case was found to have a *de novo *miss-sense mutation in the same gene [20]. Thus, approximately half of patients with BDLS features in a European population appear to exhibit *NIPBL *mutations, whereas the remaining cases may be caused by other mechanisms including X-linked genes and chromosomal imbalances. Only few cases of BDLS have been reported from countries outside Europe and North America [21,22].

## Case presentation

### Case history

**Figure 1:**
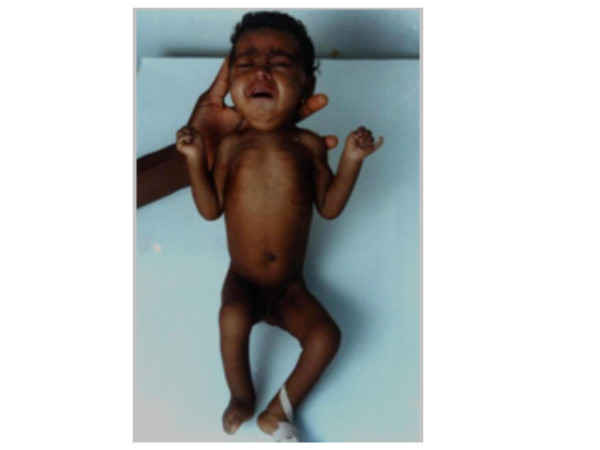
The patient external features: long curly eyelashes, low front and back hairlines, turned-up nose, down-turned angles of the mouth and thin lips, long philtrum, small lower jaw and protruding upper jaw, microcephaly, excessive body hair, short neck.

**Figure 2:**
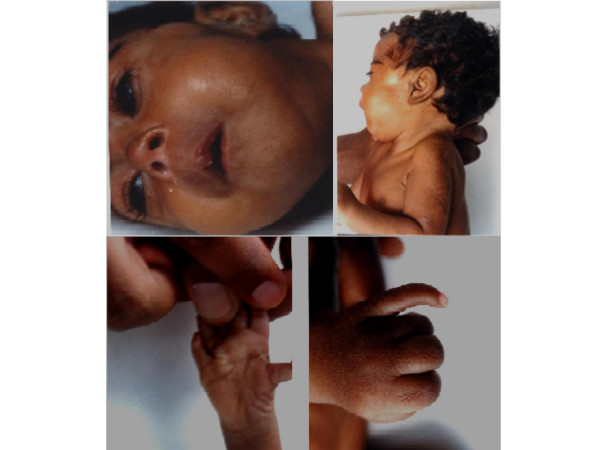
Close view of the patients facial features (a, b). The patient shows small broad hands with simian crease and proximal insertion of the thumb, and clinodactyly of the fifth finger (c, d).

A 7-month-old female infant from a Sudanese western tribe was referred as a case of malnutrition. At presentation, the patient had confluent eyebrows that appeared arched and well-defined, long curly eyelashes, low front and back hairlines, turned-up nose, down-turned angles of the mouth and thin lips, long philtrum, small lower jaw and protruding upper jaw, microcephaly, excessive body hair, short neck, and small broad hands with simian crease and proximal insertion of the thumb, and clinodactyly of the fifth finger. X-ray showed delayed bone development. She had low-pitched cry, short neck with limited movement, stiff muscle tone (unable to sit, she was still fisting), fixed flexion of both elbows, and she did not show any sign of speaking or babble (Fig. 1 and 2). Moreover she had tied tongue and thin skin. Her brain stem evoked potential test showed conductive hearing loss. She was about 27 inches in length, her weight was 3 kilograms and there were no records about her birth weight. Her skull circumference was 35 cm. Finally, she showed a number of features not typical of BDLS, including crowded ribs by chest by X-ray (distance between each rib is short). Hepatomegally was shown by ultrasonography.

### Cytogenetic analysis

Peripheral blood from the patient was subjected to short-term culture in RPMI 1640 medium for 72 hours. After metaphase arrest through exposure to Colcemide, cells were harvested, treated with hypotonic solution, and then fixed with methanol and acetic acid according to standard procedures. The harvested cells were dropped on clean slides and stained with Wright's stain, for chromosome banding [23]. The clonality criteria and the karyotypic descriptions were according to the ISCN recommendations [24]. Analysis of 11 metaphase cells showed 46, XX in all cells.

## Conclusion

Diagnosis of BDLS is dependent on the recognition of distinctive facial features [25], in addition to the physical features as pre- and postnatal growth retardation, microcephaly, severe mental retardation with speech delay, feeding problems, major malformations including limb defects, and characteristic facial features [26]. Because of the characteristic facial features the physiological findings, and the presence of a normal karyotype, the patient was diagnosed as type I BDLS [5]. She had feeding problems, which is common in BDLS, and this resulted in the malnutrition for which she was originally referred to a pediatrician. Other physiological findings supporting the diagnosis were microcephaly, excessive body hair, short neck, small broad hands with simian crease and proximal insertion of the thumb, clinodactyly of the fifth finger, short neck with limited movement, stiff muscle tone (unable to sit, she was still fisting), fixed flexion of both elbows, and delayed bone development. She had a low-pitched cry and did not show any sign of speaking or babble. The patient also showed features that were not typical of BDLS, including tied tongue, and crowded ribs. She also had hepatomegaly and decreased subcutaneous fat, which was most probably secondary to her feeding problems.

Chromosomal analysis was performed to find out if there were chromosomal imbalances or gross rearrangements of the *NIPBL *or *SMC1L1 *gene regions. The karyotype was normal. Analyses for mutations in the *NIPBL *and *SMC1L1 *genes are not currently available in Sudan and there were no funds for testing abroad. Nevertheless, based on clinical features, we believe the present patient to be the first BDLS case reported in the Sudan. Rreporting BDLS cases of different ethnic backgrounds could add nuances to the phenotypic description of the syndrome and be helpful in diagnosis. Taken together with previous reports from our laboratory [27], 28], it underscores the value of cytogenetic techniques and clinical genetic services also in low-income countries.

## Competing interests

The author(s) declare that they have no competing interests.

## Authors' contributions

ME has collected the sample, cultured it, and harvested metaphase chromosomes in the Institute of Endemic Diseases, University of Khartoum, Sudan; ME has also participated in the design and coordination of the study and drafted the manuscript. DG and TN performed the cytogenetic analysis, participated in the design of the study, and helped to draft the manuscript. AE and IF participated in the design of the study, and helped to draft the manuscript. MA diagnosed the case in Khartoum teaching hospital, supervised all the clinical investigations, participated in design of the study and helped in drafting of the manuscript. All authors agreed on the final revision of the paper.

## Pre-publication history

The pre-publication history for this paper can be accessed here:
